# The burden of physical disability among patients with newly detected leprosy in Yunnan, China, 1990–2020: A population-based, cross-sectional survey

**DOI:** 10.1371/journal.pntd.0010719

**Published:** 2022-10-11

**Authors:** Xiaohua Chen, Tie-Jun Shui

**Affiliations:** 1 Beijing Tropical Medicine Research Institute, Beijing Friendship Hospital, Capital Medical University, Beijing, China; 2 Beijing Key Laboratory for Research on Prevention and Treatment of Tropical Diseases, Capital Medical University, Beijing, China; 3 Yunnan Center for Disease Control and Prevention, Yunnan, China; Hospital Infantil de Mexico Federico Gomez, MEXICO

## Abstract

**Background:**

Physical disability is the main complication of leprosy. Although understanding the leprosy rate, prevalence, spatiotemporal distribution, and physical nerve characteristic trends is crucial for the implementation of leprosy control programs and identification of remaining challenges, these data are still unclear. We assessed physical disability trends among newly detected leprosy cases over the past 31 years in 129 counties and territories in Yunnan, China.

**Methodology/Principal findings:**

We analyzed the data of newly detected leprosy cases from the Leprosy Management Information System in Yunnan, China, from 1990–2020. All available data related to physical disability were analyzed, including demographic characteristics (sex, age, ethnicity, education level); clinical characteristics (diagnosis duration, detection mode, contact history, leprosy reaction, skin lesions, nerve lesions, disability classification); World Health Organization (WHO) leprosy physical disability indicators; and nerve and eyes, hands and feet (EHF) involvement. A total of 10758 newly diagnosed leprosy cases were identified, and 7328 (65.60%), 1179 (10.55%) and 2251 (20.15%) were associated with grade 0, 1, and 2 disability (G0D, G1D, and G2D), respectively. Male sex, older age, Han ethnicity, urban employment, a longer diagnosis duration, a contact history, greater nerve involvement, and tuberculoid-related forms of leprosy were associated with increased prevalence rates of physical disability. The rates of physical disability in newly detected leprosy cases per 1 million population decreased from 5.41, 2.83, and 8.24 in 1990 to 0.29, 0.25, and 0.54 per 1 million population in 2020, with decreases of 94.64%, 91.17%, and 93.44% in G2D, G1D and total physical disability (G1D + G2D) rates, respectively. In the same period, the proportions of G2D, G1D and total physical disability decreased from 28.02%, 14.65%, and 42.67% in 1990 to 10.08%, 11.76%, and 21.85% in 2020, with decreases of 64.03%, 19.73%, and 48.79%, respectively. Nerve thickening was more common than nerve tenderness, and claw hand, plantar insensitivity, and lagophthalmos were the most frequently reported EHF-related disabilities.

**Conclusions:**

Despite general progress in reducing the prevalence of leprosy-related physical disability, the proportion of physical disability among leprosy disease remains high, especially in specific counties. This implies that leprosy cases are being detected at a later stage and that transmission in the community still exists. Further efforts focusing on early detection are crucial for leprosy control and the elimination of the disease burden.

## Author summary

Leprosy, caused by *Mycobacterium leprae* (*M*. *leprae*), is a potentially disabling infectious disease. An understanding of the leprosy rate, prevalence, spatiotemporal distribution, and nerve characteristic trends are crucial for the implementation of leprosy control programs the identification of remaining challenges. The physical disability trends among newly detected leprosy cases were assessed over a 31-year period in Yunnan, a leprosy-endemic region in China. A total of 10758 newly diagnosed leprosy cases were reported, and 10.55% (1179) and 20.15% (2251) of patients were diagnosed with grade 1 (G1D) and grade 2 disability (G2D), respectively. Male sex, older age, Han ethnicity, urban employment, a longer diagnosis duration, a contact history, greater nerve involvement, and tuberculoid-related forms of leprosy were associated with increased prevalence rates of physical disability. Despite general progress in reducing the prevalence of leprosy-related physical disability, the proportion of those experiencing physical disability due to leprosy is still high. This implies that leprosy cases are being detected at a later stage and that transmission in the community remains. Further efforts focusing on early detection are crucial for leprosy control and the elimination of the disease burden.

## Introduction

Leprosy, caused by *Mycobacterium leprae (M*. *leprae)*, is a potentially disabling infectious disease, with over 200000 new cases reported annually worldwide. The involvement of certain peripheral nerves (neuritis) often leads to disability and devastating psychosocial consequences [1].

In 2019, 10813 leprosy cases associated with grade 2 disability (G2D) at diagnosis were reported globally, and the proportion of G2D cases among all new cases was 5.3%, corresponding to 1.2 people per million population [2]. In the absence of verifiable data, it is estimated that 3–4 million people are currently living with notable impairments or deformities due to leprosy [3].

Considering the Global Leprosy Strategy 2016–2020 targets [4], in this study, we assessed the geographic and clinical factors associated with the prevalence of physical disability. We also assessed World Health Organization (WHO) leprosy indicators of physical disability and the characteristics of nerve and eye, hands and feet (EHF) involvement associated with G2D, G1D, and total physical disability (G1D+G2D) due to leprosy over the past 31 years in Yunnan, China.

## Methods

### Ethics statement

The data for this retrospective observational study were collected from the Leprosy Management Information System in China (LEPMIS). We systematically screened the case data of patients with leprosy from local hospitals and Centers for Disease Control and Prevention (CDCs) in Yunnan, China. The study design and data analysis protocol were approved by the Ethics Committee of the Yunnan Center for Disease Control and Prevention, Yunnan, China. Individual identifying information was not available and thus informed consent was not required.

### Data sources

Patients with newly detected leprosy cases from 1990–2020 in Yunnan, China, were enrolled. The diagnosis of leprosy by clinicians met the diagnostic criteria issued by the Ministry of Health of the People’s Republic of China [5]. Patients with newly detected leprosy cases were classified as having no disability (G0D), grade 1 disability (G1D) or grade 2 disability (G2D), forming the sample for this study.

### Disability classifications, nerve involvement and the EHF score

All the patients included in this study with a confirmed diagnosis of leprosy were evaluated for physical disability level and nerve involvement according to the objective scale of physical impairment defined by the WHO [6]. The physical disability criteria were as follows: G0D: no impairment, G1D: loss of sensation, and G2D: visible impairment. Nerve involvement in leprosy was defined as signs of pain or nerve thickening upon palpation of the nerves [7]. The sum EHF score, which represents the sum of all disability scores (from 0 to 2 points) (Table 1) of the 6 sites investigated (both eyes, hands, and feet), ranged from 0 to 12 points; the EHF score is a reliable scoring tool representative of leprosy-related disability and a potentially more sensitive tool for the monitoring of disability changes and undetected disabilities than the WHO’s maximum impairment grade [8].

**Table 1:** Eye, Hand and Foot (EHF) Scores of Leprosy Cases.

Disability	Eyes	Hands and Feet
Grade 0	No eye impairment due to leprosy; no evidence of visual loss	No sensory impairment, no visible impairment
Grade 1	Eye problems due to leprosy present (irregular blink), but no vision impaired (can read fingers at six-meter distance)	Anesthesia present, but no visible deformity or damage, including muscle weakness without clawing
Grade 2	Severe visual impairments (cannot read fingers at six-meter distance), lagophthalmos, uveitis, corneal opacities	Visible impairments present, including ulcers and atrophy

### Variables

Demographic and clinical data were collected in this study. Patient basic demographic information included sex, date of birth, ethnicity, occupation, and address at the county level. Clinical characteristics included age at diagnosis, date of symptom onset, date of diagnosis, detection mode, skin lesions, nerve damage, contact history, leprosy reaction, disability classification (G0D, G1D, or G2D), Ridley-Jopling classification, and WHO classification. Diagnosis duration was defined as the time from the onset of symptoms to a confirmed diagnosis. “Early detection” was defined as a time between disease onset and diagnosis of within 2 years and the presence of G0D or G1D according to the WHO definition of leprosy disability [6].

### Spatial distribution

Population data for the study area were obtained from the National Bureau of Statistics of the People’s Republic of China. The leprosy physical disability indicator was defined according to WHO criteria [9]. The WHO leprosy indicators were as follows: (a) numbers of new cases associated with G2D, G1D or G1D + G2D at diagnosis per 1 million population, and (b) proportions of new cases associated with G2D, G1D or G1D + G2D at diagnosis. The geographical distribution of newly detected leprosy cases was mapped with ArcGIS software version 10.1 (Environmental Systems Research Institute, Inc., Redlands, CA, USA). The study period was divided into time period 1 (1990–2003: MDT) and time period 2 (2004–2020: MDT + special funding for leprosy), as described previously [10].

### Statistical analysis

Excel 2013 was used to compile the data of newly detected leprosy cases; calculate the ages of patients according to birth dates and diagnosis dates; and describe the basic demographic characteristics, time distribution trends and regional distribution characteristics of the cases. The data were subsequently analyzed using GraphPad Prism version 6 (GraphPad Software, La Jolla, CA, USA). The results of the descriptive analyses are presented as means ± standard deviations (SDs), minimum-maximum values, and medians and interquartile ranges (IQRs) for continuous variables and as counts and percentages in each category for categorical variables. The chi-square test and Fisher’s exact test were used to examine differences in the proportions of categorical variables between different groups.

## Results

### The prevalence of leprosy cases associated with physical disability

Table 2 shows the general characteristics of the study population. During the thirty-year study period, from 1990 to 2020, 11171 newly diagnosed leprosy cases were reported, and 96.3% of cases (n = 10758) were assessed for the level of physical disability at the time of diagnosis. A total of 7328 (65.60%), 1179 (10.55%) and 2251 (20.15%) were diagnosed with G0D, G1D and G2D, respectively (Table 2).

**Table 2:** The Characteristics and Prevalence Rates of Newly Detected Leprosy Cases Associated with Physical Disabilities in Yunnan, China, from 1990–2020.

Characteristics		Total		G0+1+2D		Grade 0		Grade 1		Grade 2		G1D+G2D		G1D			G2D			G1D+G2D		
														P*	PR	95%CI	P*	PR	95%CI	P*	PR	95%CI
Patient Demographic Characteristics																						
Total		11171		10758		7328		1179		2251		3430										
Gender, No.(%)	Female	3339	29.89%	3222	29.95%	2246	30.65%	365	30.96%	611	#####	976	28.45%	1[Reference]			1[Reference]			1[Reference]		
	Male	7832	70.11%	7536	70.05%	5082	69.35%	814	69.04%	1640	#####	2454	71.55%	0.838	0.988	0.8811 to 1.108	0.001	1.141	1.052 to 1.239	0.021	1.075	1.011 to 1.144
Age, Median (IQR), y		35	(26–47)	35	(26–47)	33	(25–45)	35	(27–47)	40	29–53	38	(28–51)									
Age Group, y	0–14	453	4.06%	440	4.09%	374	5.10%	34	2.88%	32	1.42%	66	1.92%	1[Reference]			1[Reference]			1[Reference]		
	15–59	9700	86.83%	9347	86.88%	6431	87.76%	1036	87.87%	1881	#####	2917	85.04%	0.001	1.665	1.209 to 2.314	<0.0001	2.871	2.068 to 4.023	<0.0001	2.08	1.671 to 2.613
	≥60	1018	9.11%	971	9.03%	523	7.14%	109	9.25%	338	#####	447	13.03%	<0.0001	2.07	1.445 to 2.981	<0.0001	4.981	3.558 to 7.031	<0.0001	3.072	2.446 to 3.888
Ethnic Group	Han	5264	47.12%	5085	47.27%	3287	44.86%	643	54.54%	115	#####	1798	52.42%	<0.0001	1.397	1.256 to 1.554	<0.0001	1.219	1.134 to 1.310	<0.0001	1.229	1.163 to 1.299
	Minor ethnics	5907	52.88%	5673	52.73%	4041	55.14%	536	45.46%	1096	#####	1632	47.58%	1[Reference]			1[Reference]			1[Reference]		
Occupation	Urban	10254	91.79%	9905	92.07%	6668	90.99%	1099	93.21%	2198	#####	3237	94.37%	0.012	1.309	1.060 to 1.624	<0.0001	1.661	1.399 to 1.981	<0.0001	1.444	1.275 to 1.644
	City	918	8.22%	854	7.94%	660	9.01%	80	6.79%	113	5.02%	193	5.63%	1[Reference]			1[Reference]			1[Reference]		
Patient Clinic Characteristics																						
Diagnosis Duration, y	< 2	7168	64.17%	6934	64.45%	5295	72.26%	747	63.36%	892	#####	1639	47.78%	1[Reference]			1[Reference]			1[Reference]		
	2~4.9	2924	26.17%	2787	25.91%	1624	22.16%	321	27.23%	842	#####	1163	33.91%	<0.0001	1.335	1.183 to 1.505	<0.0001	2.368	2.182 to 2.570	<0.0001	1.765	1.661 to 1.876
	5–9.9	687	6.15%	656	6.10%	273	3.73%	79	6.70%	304	#####	383	11.17%	<0.0001	1.815	1.472 to 2.217	<0.0001	3.654	3.306 to 4.024	<0.0001	2.47	2.282 to 2.663
	≥10	392	3.51%	381	3.54%	136	1.86%	32	2.71%	213	9.46%	245	7.14%	0.013	1.541	1.111 to 2.091	<0.0001	4.233	3.803 to 4.679	<0.0001	2.72	2.487 to 2.954
Detection Mode	Passive Case Finding	7152	64.02%	6937	64.48%	4736	64.63%	795	67.43%	1406	#####	2201	64.17%	1[Reference]			1[Reference]			1[Reference]		
	Active Case Finding	4019	35.98%	3821	35.52%	2592	35.37%	384	32.57%	845	#####	1129	35.83%	0.065	0.898	0.8013 to 1.005	0.063	1.074	0.9968 to 1.157	0.142	0.956	0.9007 to 1.015
	Self-reported illness	2299	20.58%	2231	20.74%	1594	21.75%	227	19.25%	411	#####	638	18.60%	1[Reference]			1[Reference]			1[Reference]		
	Out-patient clinic finding	3027	27.10%	2931	27.24%	2066	28.19%	352	29.86%	513	#####	865	25.22%	0.052	1.168	1.000 to 1.364	0.63	0.97	0.8644 to 1.090	0.477	1.032	0.9474 to 1.126
	Other-reported illness	1826	16.35%	1775	16.50%	1076	14.68%	216	18.32%	482	#####	698	20.35%	0.001	1.341	1.129 to 1.592	<0.0001	1.509	1.347 to 1.691	<0.0001	1.376	1.261 to 1.502
	Contact examination	1286	11.51%	1259	11.70%	975	13.31%	135	11.45%	149	6.62%	284	8.28%	0.001	5.777	1.639 to 21.15	0.04	2.187	1.036 to 4.784	2E-04	2.848	1.505 to 5.585
	Focus Survey	360	3.22%	345	3.21%	246	3.36%	3	2.54%	66	2.93%	96	2.80%	0.006	5.163	1.420 to 19.40	4E-04	3.49	1.628 to 7.736	<0.0001	3.544	1.846 to 7.035
	Group examination	114	1.02%	113	1.05%	93	1.27%	2	0.17%	6	0.27%	8	0.23%	1[Reference]			1[Reference]			1[Reference]		
	Clue investigation	1965	17.59%	1855	17.24%	1089	14.86%	204	17.30%	574	#####	778	22.68%	<0.0001	7.494	2.133 to 27.37	<0.0001	5.695	2.732 to 12.33	<0.0001	5.261	2.798 to 10.26
	Leprosy Elimination Campaign (LEC)	137	1.23%	127	1.18%	94	1.28%	10	0.85%	23	1.02%	33	0.96%	0.035	4.567	1.166 to 18.28	0.005	3.244	1.429 to 7.530	4E-04	3.281	1.633 to 6.750
	Other ways	157	1.41%	123	1.14%	95	1.30%	3	0.25%	27	1.20%	30	0.87%	>0.9999	1.454	0.2964 to 7.168	0.001	3.652	1.632 to 8.376	0.001	3.03	1.496 to 6.277
Contact History	Unknown	3572	32.98%	3349	31.13%	2253	30.75%	349	29.60%	747	#####	1096	31.95%	1[Reference]			1[Reference]			1[Reference]		
	Present	7599	68.02%	7408	68.87%	5075	69.25%	830	70.40%	1504	#####	2334	68.05%	0.434	1.048	0.9334 to 1.178	0.031	0.918	0.8509 to 0.9913	0.211	0.963	0.9077 to 1.021
	Within family	3404	30.47%	3327	30.93%	2403	32.79%	379	32.15%	545	#####	924	26.94%	1[Reference]			1[Reference]			1[Reference]		
	Out of family	4195	37.55%	4082	37.94%	2672	36.46%	451	38.25%	959	#####	1410	41.11%	0.368	1.06	0.9341 to 1.203	<0.0001	1.429	1.302 to 1.569	<0.0001	1.244	1.161 to 1.333
Leprosy Reaction	Absent	10841	97.05%	10439	97.03%	7139	97.42%	1099	93.21%	2201	#####	3300	96.21%	1[Reference]			1[Reference]			1[Reference]		
	Present	330	2.98%	319	3.97%	189	2.58%	80	6.79%	50	2.22%	130	3.79%	<0.0001	2.229	1.830 to 2.681	0.395	0.888	0.6876 to 1.129	8E-04	1.289	1.120 to 1.467
Skin Lesion	0	448	4.01%	440	4.09%	229	3.13%	44	3.73%	167	7.42%	211	6.15%	0.062	1.378	0.9943 to 1.895	<0.0001	1.964	1.664 to 2.310	<0.0001	1.661	1.451 to 1.895
	1	1164	10.42%	1136	10.56%	808	11.03%	107	9.08%	221	9.82%	328	9.56%	1[Reference]			1[Reference]			1[Reference]		
	(2–5)	3087	27.63%	3044	28.30%	2003	27.33%	326	27.65%	715	#####	1041	30.35%	0.085	1.197	0.9773 to 1.470	0.002	1.225	1.074 to 1.400	0.001	1.184	1.069 to 1.315
	Over 5	5749	51.46%	5643	52.45%	3923	53.53%	670	56.83%	1071	#####	1741	50.76%	0.022	1.247	1.032 to 1.512	>0.9999	0.999	0.8797 to 1.137	0.216	1.065	0.9653 to 1.177
	Missing data	723	6.47%	495	4.60%	176	2.40%	32	2.71%	77	3.42%	109	3.18%									
Nerve thickening and/or tenderness on palpation	0	1138	10.19%	1114	10.36%	970	13.24%	53	4.50%	91	4.04%	144	4.20%	1[Reference]			1[Reference]			1[Reference]		
	1	2173	19.45%	2130	19.80%	1455	19.86%	238	20.19%	437	#####	675	19.68%	<0.0001	2.713	2.041 to 3.618	<0.0001	2.693	2.180 to 3.335	<0.0001	2.452	2.083 to 2.894
	2	7425	66.47%	7318	68.02%	4760	64.96%	871	73.88%	1687	#####	2558	74.58%	<0.0001	2.986	2.287 to 3.911	<0.0001	3.051	2.502 to 3.734	<0.0001	2.704	2.319 to 3.164
	Missing data	435	3.89%	196	1.82%	143	1.95%	17	1.44%	36	1.60%	53	1.55%									
Ridley-Jopling Classification	LL	1509	13.51%	1423	13.23%	1043	14.23%	157	13.32%	223	9.91%	380	11.08%	1[Reference]			1[Reference]			1[Reference]		
	BL	4525	40.51%	4368	40.60%	3102	42.33%	549	46.56%	717	#####	1266	36.91%	0.099	1.149	0.9759 to 1.357	0.38	1.066	0.9312 to 1.222	0.104	1.085	0.9852 to 1.198
	BB	1080	9.67%	1053	9.79%	721	9.84%	121	10.26%	211	9.37%	332	9.68%	0.432	1.098	0.8812 to 1.367	0.004	1.285	1.086 to 1.520	0.009	1.181	1.043 to 1.336
	BT	2661	23.82%	2581	23.99%	1597	21.79%	230	19.51%	754	#####	984	28.69%	0.697	0.962	0.7968 to 1.163	<0.0001	1.821	1.596 to 2.081	<0.0001	1.428	1.294 to 1.578
	TT	1214	10.87%	1162	10.80%	719	9.81%	110	9.33%	333	#####	443	12.92%	0.947	1.014	0.8080 to 1.271	<0.0001	1.797	1.550 to 2.085	<0.0001	1.428	1.275 to 1.599
	I	165	1.48%	164	1.52%	142	1.94%	12	1.02%	10	0.44%	22	0.64%	0.069	0.596	0.3388 to 1.024	3E-04	0.374	0.2029 to 0.6717	1E-04	0.502	0.3351 to 0.7368
	Missing data	17	0.15%	7	0.07%	4	0.05%	0	0.00%	3	0.13%	3	0.09%									
WHO Classification	Multibacillary form	7574	67.80%	7299	67.85%	5193	70.87%	875	74.22%	1231	#####	2106	61.40%	1[Reference]			1[Reference]			1[Reference]		
	Paucibacillary form	3580	32.15%	3452	32.09%	2131	29.08%	304	25.78%	1017	#####	1321	38.51%	0.02	0.866	0.7662 to 0.9773	<0.0001	1.686	1.570 to 1.810	<0.0001	1.326	1.254 to 1.402
	Missing data	17	0.15%	7	0.07%	4	0.05%	0	0.00%	3	0.13%	3	0.09%									

The distribution of cases according to sex demonstrated that 72.86% of the cases with G2D were males, who had a 1.141-fold higher prevalence of physical disability due to leprosy than females (p = 0.014). Regarding age groups, 83.56% and 15.02% of the reported patients with G2D were 15–59 years old and over 60 years old, respectively, and these patients had a 2.871- and 4.981-fold higher prevalence of disability than patients under 15 years old, respectively (p <0.0001 and <0.0001, respectively). Regarding ethnic groups, Han ethnicity was associated with a 1.219-fold higher prevalence of disability than patients belonging to minor ethnicity groups. Similar trends were also found for total physical disability (G1D+G2D) and G1D.

Regarding the diagnosis duration, 2–5 years, 5–10 years and over 10 years were associated with 2.368-, 3.652- and 4.233-fold higher G2D prevalence rates than a duration of less than 2 years, respectively (P<0.0001, <0.0001, and <0.0001, respectively). Regarding the detection mode, other modes were associated with a 1.509-fold higher prevalence of G2D than self-reporting (P<0.0001). In detail, contact examination, Leprosy Elimination Campaign (LEC), disease focus investigation, and suspected disease clue investigation were associated with 2.187-, 3.244-, 3.490-, and 5.695-fold higher prevalence rates of G2D than group examination, respectively (P<0.05). However, there was no association between passive and active detection modes and the development of G2D (p = 0.0505). Regarding traceable infectious sources, infection from sources outside the family was associated with a 1.429-fold higher prevalence of G2D than infection from sources within the family (P<0.0001).

Regarding skin lesions, 2–5 skin lesions and 0 skin lesions were associated with 1.225- and 1.964-fold higher prevalence rates of G2D than 1 skin lesion, respectively (P = 0.0023 and <0.0001). Regarding nerve lesions, 1 nerve lesion and 2 nerve lesions were associated with 2.693- and 3.051-fold higher prevalence rates of G2D than no nerve lesions (P<0.0001, and <0.0001). Regarding the Ridley-Jopling classification, the mid-borderline (BB), mid-borderline (BT) and tuberculoid (TT) forms were associated with 1.285-, 1.821- and 1.797-fold higher prevalence rates of G2D than the lepromatous (LL) form of leprosy, respectively (P = 0.0040, <0.0001, and <0.0001). Regarding the WHO classification, paucibacillary (PB) form was associated with a 1.686-fold higher prevalence of G2D than the multibacillary (MB) form of leprosy (P<0.0001).

### Temporal distributions of WHO leprosy indicators of physical disability

**Fig 1:**
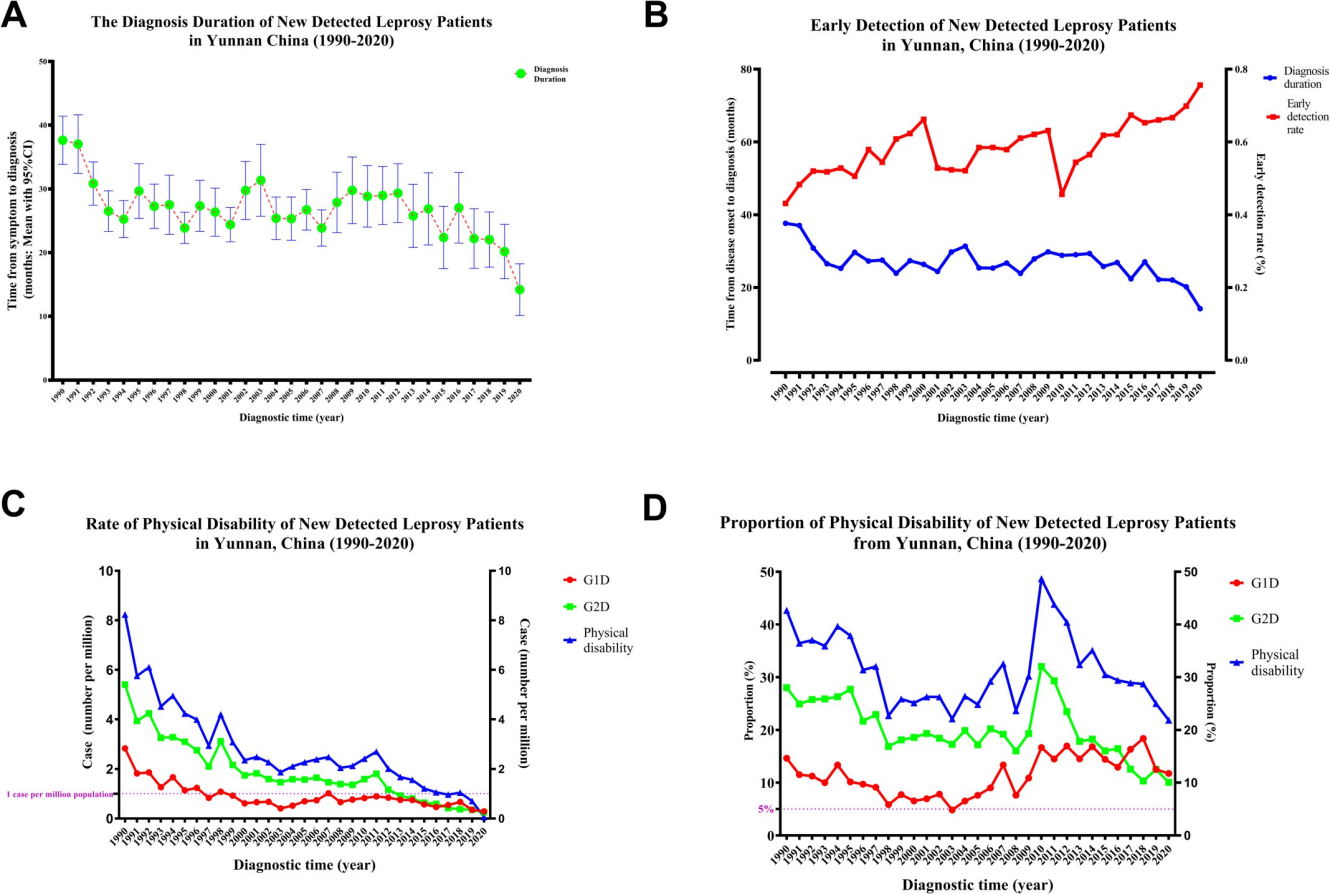
Diagnostic Durations and WHO Leprosy Indicators of Physical Disability in Yunnan, China, 1990–2020.

The diagnostic durations and rates of early detection are shown in Fig 1A and 1B. The diagnostic duration decreased from 37.63 months in 1990 to 14.19 months in 2020 (S1 Table and Fig 1A). During the same period, the rate of early detection increased from 43.10% in 1990 to 75.63% in 2020 (S1 Table and Fig 1B).

The rates and proportions of physical disabilities in newly detected cases of leprosy are shown in Fig 1C and 1D. With the dramatic decreases in the rates of newly detected leprosy cases, the rates of G2D, G1D, and G1D + G2D (cases per 1 million population) also decreased from 1990 to 2020 (S2 Table and Fig 1C). The rates of G2D, G1D, and G1D + G2D among patients with newly detected leprosy cases decreased from 5.41, 2.83, and 8.24 per 1 million population in 1990 to 0.25, 0.29, and 0.54 per 1 million population in 2020, respectively. However, the proportion of new cases with physical disability at diagnosis remained over 20% (S2 Table and Fig 1C). The proportion of new cases with total physical disability initially decreased from 42.67% in 1990 to 30.22% in 2009, increased to the highest rate (48.68%) in 2010, and finally decreased to 21.85% in 2020. The proportion of cases associated with G2D showed a similar trend, ranging from 10.08% to 32.02%, with the highest and lowest rates observed in 2010 and 2020, respectively. The proportion of cases associated with G1D ranged from 4.82% to 18.39%, with the highest and lowest rates observed in 2003 and 2018, respectively (S2 Table and Fig 1D).

### Spatial distributions of WHO leprosy indicators of physical disability

**Fig 2:**
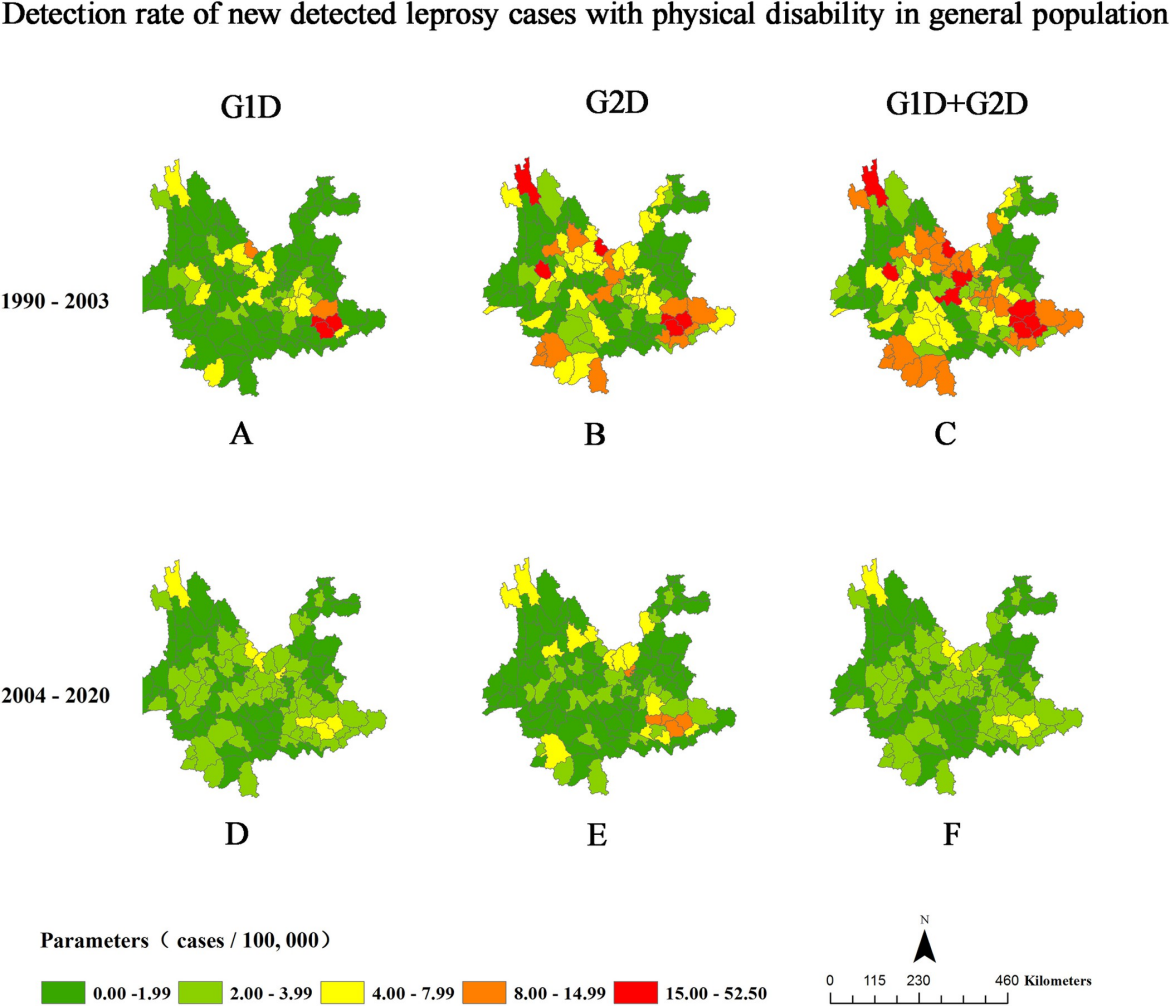
Proportions of Physical Disability Among Newly Detected Leprosy Cases in Yunnan, China, 1990–2020. The proportions of newly detected leprosy cases associated with G1D (A, D), G2D (B, E), and total physical disability (C, F) in the general population in time period 1 (1990–2003) (A, B, C) and time period 2 (2004–2020) (D, E, F). Map from Naive Map developed in AMAP with data from the National Catalogue Service For Geographic Information. https://www.naivemap.com/admin-cn-downloader/.

**Fig 3:**
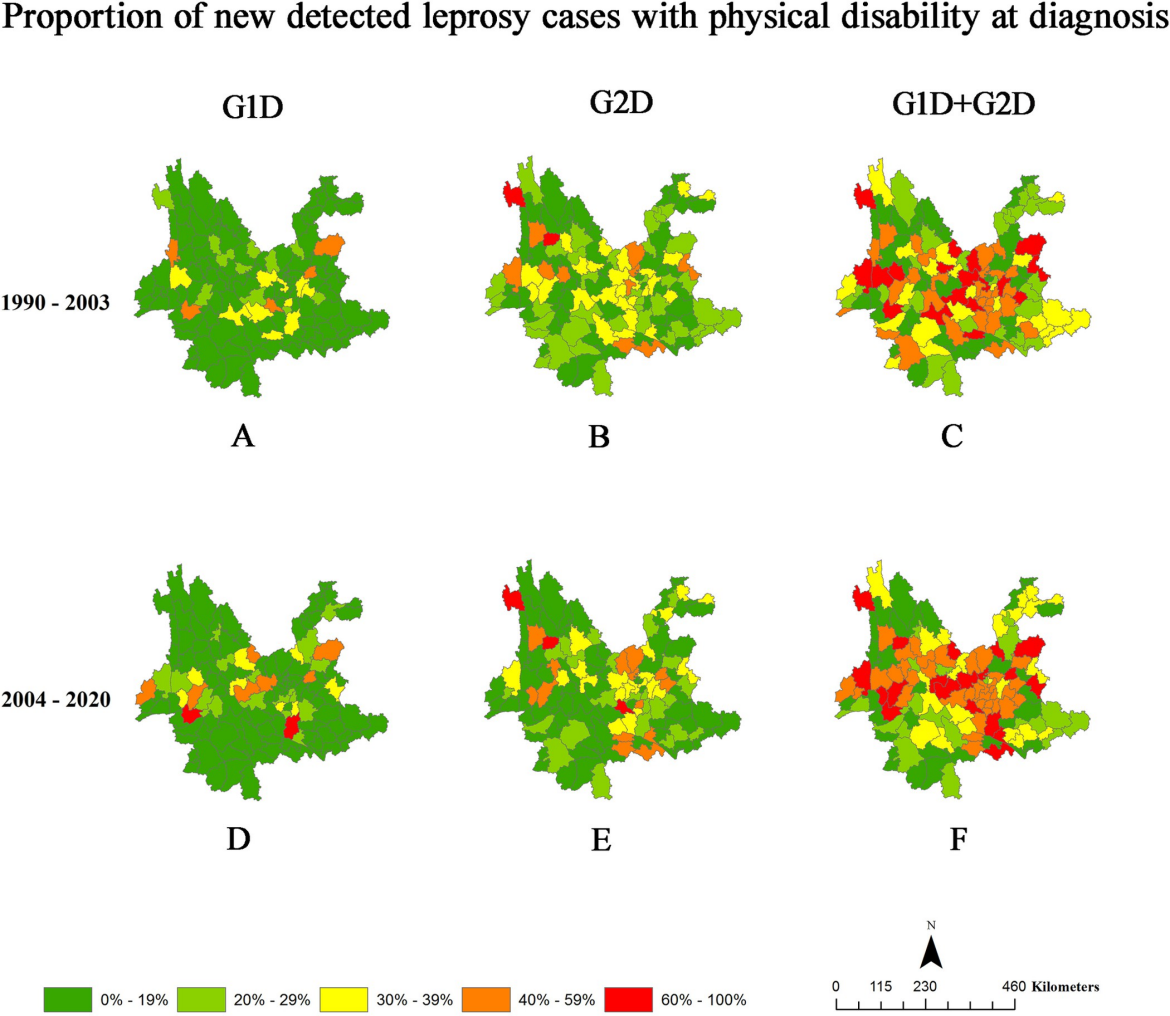
Detection Rates of Physical Disability Among Newly Detected Leprosy Cases in Yunnan, China, 1990–2020. The detection rates of G1D (A, D), G2D (B, E), and total physical disability (C, F) among newly detected leprosy cases in the general population in time period 1 (1990–2003) (A, B, C) and time period 2 (2004–2020) (D, E, F). Map from Naive Map developed in AMAP with data from the National Catalogue Service For Geographic Information. https://www.naivemap.com/admin-cn-downloader/.

Figs 2 and 3 and S3 Table show the spatial distributions of G1D, G2D, and total physical disability among newly detected leprosy cases in Yunnan, China. A total of 98.45% (127/129) of counties in Yunnan reported leprosy cases during the study period, and 96.85% (123/127) of counties registered new cases of leprosy associated with G2D over the study period.

Regarding the G2D rate per 1 million population, 29.27% (36/123) of counties with new cases had very low rates (0.0–1.9), 16.26% (20/123) had low rates (2.0–3.99), 27.64% (34/123) had moderate rates (4.0–7.99), 17.89% (22/123) had high rates (8.0–14.99), and 8.94% (11/123) had very high rates (≥15 cases per 1 million population) (S3 Table and Fig 2). Regarding the proportion of new leprosy cases associated with G2D at diagnosis, 35.77% (44/123) of counties had a low proportion (0.00%-19.99%), 53.66% (66/123) of counties had a moderate proportion (20.00%-39.99%), 8.94% (11/123) of counties had a high proportion (40.00%-60.00%), and 1.62% (2/123) of counties had a very high proportion (60.00%-100.00%) (S3 Table and Fig 3).

Compared with that in time period 1, the number of counties with high and very high rates (over 8 cases per 1 million population) of G2D decreased dramatically from 20 to 4 counties, while the number of counties with high and very high proportions (over 40.00%) of G2D decreased only slightly from 21 to 19 counties during time period 2 (Figs 2 and 3, and S3 Table).

### Characteristics of nerve and EHF involvement

At the time of diagnosis. A total of 69.99% (7819/11171) of cases were assessed for nerve (Table 3) and EHF involvement (Table 4). Nerve thickening and nerve tenderness examined by nerve palpation were evaluated in the study population, and the positivity rate for nerve thickening (13.29%, 8314/62552) was higher than that for nerve tenderness (3.96%, 2477/7819) (P<0.05) (Table 5). The most common thickened nerves were the ulnar (1679/7819, 21.47%; and 1651/7819, 21.12% for the right and left sides, respectively), common fibular (1204/7819, 15.40%; and 1189/7819, 15.21% for the right and left sides, respectively) and greater auricular (822/7819, 10.51%; and 824/7819, 10.54% for the right and left sides, respectively) nerves (Table 5). The most common nerves with tenderness were also the ulnar (501/7819, 6.41%; and 513/7819, 6.56% for the right and left sides, respectively), common fibular (355/7819, 4.54%; and 348/7819, 4.45% for the right and left sides, respectively) and greater auricular (208/7819, 2.66%; and 207/7819, 2.65% for the right and left sides, respectively) nerves (Table 3). Regarding the grade of disability, the positivity rate for G1D was higher than that for G2D (Table 3).

**Table 3:** The Characteristics of Nerve Involvement Among Leprosy Patients in Yunnan, China, 1990–2020.

Characteristics	Nerves involved		Grade 0		Grade 1		Grade 2		Total	
			N	%	n	%	n	%	n	%
		Total (n, %)	4836	61.85%	915	11.70%	1714	21.92%	7819	100%
Nerve Thickness	Greater auricular	Right	490	10.08%	138	15.08%	174	10.15%	822	10.51%
		Left	491	10.10%	137	14.97%	172	10.04%	824	10.54%
	Supra-orbital	Right	81	1.67%	36	3.93%	42	2.45%	168	2.15%
		Left	87	1.79%	34	3.72%	39	2.28%	169	2.16%
	Ulnar	Right	928	19.08%	266	29.07%	441	25.73%	1679	21.47%
		Left	902	18.55%	278	30.38%	421	24.56%	1651	21.12%
	Common fibular	Right	687	14.13%	207	22.62%	281	16.39%	1204	15.40%
		Left	663	13.63%	219	23.93%	275	16.04%	1189	15.21%
	Median	Right	45	0.93%	29	3.17%	66	3.85%	143	1.83%
		Left	43	0.88%	28	3.06%	61	3.56%	135	1.73%
	Tibial	Right	45	0.93%	23	2.51%	36	2.10%	104	1.33%
		Left	48	0.99%	26	2.84%	37	2.16%	111	1.42%
	Radial	Right	16	0.33%	9	0.98%	23	1.34%	48	0.61%
		Left	17	0.35%	9	0.98%	21	1.23%	47	0.60%
	Facial	Left	4	0.08%	1	0.11%	3	0.18%	9	0.12%
		Right	6	0.12%	2	0.22%	2	0.12%	11	0.14%
Nerve Tenderness	Greater auricular	Right	130	2.67%	32	3.50%	40	2.33%	208	2.66%
		Left	120	2.47%	31	3.39%	47	2.74%	207	2.65%
	Supra-orbital	Right	32	0.66%	13	1.42%	14	0.82%	67	0.86%
		Left	35	0.72%	15	1.64%	15	0.88%	73	0.93%
	Ulnar	Right	258	5.31%	86	9.40%	137	7.99%	501	6.41%
		Left	264	5.43%	90	9.84%	135	7.88%	513	6.56%
	Common fibular	Right	187	3.85%	63	6.89%	86	5.02%	355	4.54%
		Left	176	3.62%	67	7.32%	84	4.90%	348	4.45%
	Median	Right	18	0.37%	10	1.09%	18	1.05%	47	0.60%
		Left	18	0.37%	8	0.87%	16	0.93%	43	0.55%
	Tibial	Right	20	0.41%	7	0.77%	7	0.41%	34	0.44%
		Left	19	0.39%	7	0.77%	9	0.53%	35	0.45%
	Radial	Right	8	0.17%	3	0.33%	7	0.41%	18	0.23%
		Left	9	0.19%	4	0.44%	6	0.35%	19	0.24%
	Facial	Right	2	0.04%	0	0.00%	0	0.00%	2	0.03%
		Left	4	0.08%	2	0.22%	1	0.06%	7	0.09%

**Table 4:** Characteristics of Disabilities of the EHF Among Leprosy Patients in Yunnan, China, 1990–2020.

	Total EHF (n, %)	Right (n, %)		Left (n, %)		Total	
		7819	50.00%	7819	50.00%	15638	100.00%
Eye	Total eye	189	2.42%	196	2.51%	377	2.41%
	Insensitivity	48	0.61%	47	0.60%	94	0.60%
	Lagophthalmos	56	0.72%	56	0.72%	109	0.70%
	Ectropion	8	0.10%	8	0.10%	16	0.10%
	Trichiasis	3	0.04%	3	0.04%	6	0.04%
	Exposure keratitis	12	0.15%	12	0.15%	24	0.15%
	Iritis (Iridocyclitis)	17	0.22%	18	0.23%	35	0.22%
	Decrease of vision	39	0.50%	44	0.56%	79	0.51%
	Blindness	6	0.08%	8	0.10%	14	0.09%
	EHF = 0	7680	98.22%	7671	98.11%	/	/
	EHF = 1	41	0.52%	40	0.51%	/	/
	EHF = 2	98	1.25%	108	1.38%	/	/
Hand	Total hand	816	10.44%	784	10.03%	1561	9.98%
	Insensitivity	338	4.32%	353	4.51%	677	4.33%
	Claw hand	261	3.34%	238	3.04%	485	3.10%
	Ape hand	49	0.63%	45	0.58%	91	0.58%
	Wrist drop	6	0.08%	7	0.09%	13	0.08%
	Keratosis and chapped wound	44	0.56%	35	0.45%	77	0.49%
	Palmar ulcer	24	0.31%	11	0.14%	33	0.21%
	Stiff joint	45	0.58%	40	0.51%	84	0.54%
	Absorption	49	0.63%	55	0.70%	101	0.65%
	EHF = 0	7230	92.47%	7245	92.66%	/	/
	EHF = 1	246	3.15%	259	3.31%	/	/
	EHF = 2	343	4.39%	315	4.03%	/	/
Foot	Total foot	585	7.48%	595	7.51%	1150	7.35%
	Insensitivity	379	4.85%	388	4.96%	744	4.76%
	Foot drop	13	0.17%	9	0.12%	21	0.13%
	Skin chapped wound	51	0.65%	43	0.55%	93	0.59%
	Simple plantar ulcer	78	1.00%	87	1.11%	162	1.04%
	Complex plantar ulcer	19	0.24%	19	0.24%	36	0.23%
	Clawed toes slight absorption	38	0.49%	44	0.56%	82	0.52%
	Equinus	5	0.06%	3	0.04%	8	0.05%
	Amputation	2	0.03%	2	0.03%	4	0.03%
	EHF = 0	7347	93.96%	7324	93.67%	/	/
	EHF = 1	313	4.00%	325	4.16%	/	/
	EHF = 2	159	2.03%	170	2.17%	/	/
EHF Total	EHF = 0	6854	87.66%	/	/	/	/
	EHF = 1	102	1.30%	/	/	/	/
	EHF = 2	317	4.05%	/	/	/	/
	EHF = 3	53	0.68%	/	/	/	/
	EHF = 4	261	3.34%	/	/	/	/
	EHF = 5	24	0.31%	/	/	/	/
	EHF = 6	103	1.32%	/	/	/	/
	EHF = 7	11	0.14%	/	/	/	/
	EHF = 8	53	0.68%	/	/	/	/
	EHF = 9	7	0.09%	/	/	/	/
	EHF = 10	17	0.22%	/	/	/	/
	EHF = 11	5	0.06%	/	/	/	/
	EHF = 12	12	0.15%	/	/	/	/

**Table 5:** Diagnosis Durations and Physical Disabilities Associated with Leprosy in Yunnan, China, 1990–2020.

Diagnosis duration, y	Grade 0 (n, %)		Grade 1 (n, %)		Grade 2 (n, %)		Total (n, %)	
< 2y	5294	76.37%	746	10.76%	892	8.29%	6932	64.44%
2~4.99y	1624	58.25%	322	11.55%	842	30.20%	2788	25.92%
5–9.99y	274	41.64%	79	12.01%	305	46.35%	658	6.12%
≧10y	136	35.79%	32	8.42%	212	55.79%	380	3.53%
Total	7328	68.12%	1179	10.96%	2251	20.92%	10758	100.00%

Table 4 shows the total disabilities of the EHF. Among disabilities of the EHF, the hands were the most affected (1561/15638, 9.98%), followed by the feet (1150/15638, 7.35%) and the eyes (377/15638, 2.41%). Table 4 shows the deformities of the EHF. The most frequent eye disabilities were lagophthalmos (295/15638, 0.70%), insensitivity (94/15638, 0.60%), and decreased visual ability (79/15638, 0.51%). The most frequent hand disabilities were palmar insensitivity (677/15638, 4.33%) and claw hand (485/15638, 3.10%). The most frequent foot disabilities were palmar insensitivity (744/15638, 4.76%) and simple plantar ulceration (162/15638, 1.04%).

Table 5 shows the diagnosis duration of and physical disabilities associated with leprosy. A total of 76.37% (5294/6932) of leprosy cases associated with G0D were diagnosed within 2 years. With the prolongation of the diagnostic duration, the proportions gradually decreased, with proportions of 58.25% (1624/2788), 41.64% (274/658) and 35.79% (136/380) for diagnosis durations of 2–4.99 years, 5–9.99 years, and over 10 years, respectively. In contrast, the proportions of leprosy cases associated with G2D diagnosed within 2 years, 2–4.99 years, 5–9.99 years and over 10 years were 8.92% (892/6932), 30.20% (842/2788), 46.35% (305/658), and 55.79% (212/380), respectively.

## Discussion

This survey found that 31.88% (3430/10758) of newly detected leprosy cases had different degrees of disability. In 2018, the prevalence of leprosy-related disability in Yunnan (10.34%) was lower than that in China (19.0%) [11]. This may be due to the effective multistrategy for leprosy control in Yunnan, China [10].

The WHO leprosy-related physical disability indicators are commonly used by control programs to monitor and evaluate the epidemiological situation of leprosy, reveal the changes in the transmission chain and form conclusions regarding the quality of the health care services [12]. This study evaluated the trends of the WHO physical disability leprosy indicators in Yunnan, China, using historical data from a period of 31 years. Our data showed that the rates of physical disabilities among patients with newly detected leprosy cases per 1 million population decreased dramatically by 94.64%, 91.17%, and 93.44% for G2D, G1D and total physical disability, respectively. In the same period, the proportions of G2D, G1D and total physical disability decreased by 64.03%, 19.73%, and 48.79%, respectively. Despite the relatively low rate of physical disability evaluation among leprosy patients per 1 million population, the data revealed a high proportion of physical disability at diagnosis among newly detected leprosy cases.

The global strategy for the control of leprosy from 2011 to 2015 aimed to reduce the rate of new cases with G2D worldwide by more than 35% by the end of 2015 compared with the baseline at the end of 2010 [7]. Our findings revealed that the proportions of G2D among newly detected leprosy cases were 32.02% in 2010 and 16.04% in 2015, with a decrease of 49.91% in Yunnan, China, which was over the 35% target considering the baseline at the end of 2010.

The global strategy for the control of leprosy from 2016 to 2020 aimed to reduce the number of newly diagnosed leprosy patients with visible deformities to less than 1 per million population [4]. The rate of new leprosy cases with G2D per 1 million population is an impact indicator that reflects delayed diagnosis. According to the current study, this indicator has been less than 1 case per 1 million population since 2013 in Yunnan, China, indicating a high level of early detection of leprosy cases in the study region. The proportion of G2D cases among newly detected leprosy cases, another indicator, also reflects a delay in diagnosis. The G2D proportion ranges from 1.8% in the Federated States of Micronesia to 18.6% in China and 42.1% in Somalia [13]. The global average of this indicator is 6.7%. Generally, figures above 5% are considered to reflect delayed case detection. However, in this study, the prevalence rates of G2D and total physical disability were 10.08% and 21.85% in 2020 in Yunnan, China, implying that delayed leprosy diagnosis is still a problem in the study region.

The high proportion of new leprosy cases associated with G2D at diagnosis may reflect operational problems and barriers in access to health care services and supports evidence that the transmission chain is being maintained in the community since, in general, leprosy patients with visible disabilities have advanced forms of the disease (e.g., MB leprosy) [12]. In Yunnan, China, 17.07% (21/123) of counties had a G2D proportion of over 40.00% from 1990–2003, and 15.45% (19/123) of counties had a G2D proportion of over 40.00% from 2004–2020. These results indicates that the transmission chain is still active in communities in certain regions.

In this study, nerve thickening has a higher positivity rate than nerve tenderness (13.29% vs. 3.96%). Regarding disabilities of the EHF, the hands (9.98%) were more affected by disabilities than the feet (7.35%) or eyes (2.41%). Claw hand, plantar insensitivity and simple plantar ulceration were the most frequent disabilities in the hands and feet. In our previous studies, nerve enlargement in the peripheral upper limbs detected by ultrasound [14] and claw hand were the most frequently reported symptoms in leprosy patients [15]. Lagophthalmos, insensitivity and decreased visual ability were the most frequent disabilities of the eyes. It has been reported that when neuritis occurs in individuals who do not receive proper treatment, the condition may become chronic, leading to the development of characteristic physical disabilities associated with leprosy [16]. In addition, medical personnel are not very familiar with leprosy because of its low prevalence, which could lead to a delay in diagnosis. During this time, peripheral nerve damage develops, leading to disability [17]. Thus, understanding the characteristics of nerve involvement would help medical personnel identify leprosy in the early stage, avoiding a delayed diagnosis and preventing irreversible deformities. We also observed that although G0D was generally associated with a shorter diagnosis duration among newly detected leprosy cases and G2D was mainly associated with a longer diagnosis duration, some cases rapidly progressed to irreversible deformity within 2 years after symptom onset, while other cases did not progress to physical disability even with a disease duration of over 10 years. This may imply that there are risk factors in addition to early detection that influence physical disability.

Our study has some limitations. A proportion of leprosy patients had an unknown degree of physical disability, which may have had little effect on the WHO leprosy indicators used in this study. In addition, physical disabilities remained after completion of multidrug therapy (MDT) and frequently recurred in an endemic area in Brazil [18]. Systematic follow-up of patients after treatment completion should be assessed in the study area in the future. The coronavirus disease 2019 (COVID-19) pandemic had a significant impact on health services in all countries, and leprosy programs were clearly affected, as evidenced by the substantial reduction in the number of cases detected and reported by countries in 2020 [19]; this may be a potential source of bias in 2020 leprosy data from Yunnan, China.

## Conclusion

In Yunnan, China, the rate of leprosy-related physical disability per 1 million population has decreased dramatically. Despite general progress in reducing the prevalence of physical disability associated with leprosy, the proportion of leprosy-related physical disability remains high, and leprosy-related physical disability still imposes a substantial burden on patients and societies. Strengthening health systems to improve early case detection and improving the quality of leprosy care, including prompt and accurate diagnostics, early initiation of treatment, and routine follow-up, are priorities. Counties for which the leprosy-related physical disability burden is high should investigate the reasons for the high burden and address them appropriately.

## Supporting information

S1 Table Diagnostic Durations and Early Detection Rates of Newly Detected Leprosy Cases in Yunnan, China, 1990–2020. (XLS)

S2 Table Rates and Proportions of Physical Disabilities Among Newly Detected Leprosy Cases in Yunnan, China, 1990–2020. (XLS)

S3 Table WHO Leprosy Indicators of Physical Disability Among Newly Detected Leprosy Cases in Yunnan, China, 1990–2020. (XLS)
